# Applying conscientiousness index: a tool to explore medical students’ professionalism in Indonesia

**DOI:** 10.5116/ijme.5780.9916

**Published:** 2016-07-14

**Authors:** Wolter Prakarsa Jaya, Elisabeth Rukmini

**Affiliations:** 1 School of Medicine, Atma Jaya Catholic University of Indonesia, Indonesia; 2 Medical Education Unit, School of Medicine, Atma Jaya Catholic University of Indonesia, Indonesia

**Keywords:** Conscientiousness Index (CI), professionalism, assessment, medical education

## Abstract

**Objectives:**

This study was aimed to describe lecturers’ perspective concerning the suitable Conscientiousness Index (CI) components and implementations, as well as to compare the CI scores in year 1–4 student batches.

**Methods:**

Components were formulated from objective measurements based on interviews with 12 faculty members. The components include: attendance, adherence to rules, evaluative feedback submissions, performance in assignments and clinical skills, assignment submissions, volunteerism, accomplishments, and general misconducts. The scores were collected from year 1-4 pre-clinical medical students (N=144) during the first semester of 2014-2015. Final interviews were conducted with 9 faculty members. Quantitative analysis was performed using Kruskal-Wallis and Mann-Whitney test. Qualitative analysis was performed using content analysis.

**Results:**

Using Kruskal-Wallis test, significant difference was found in the CI scores among all years (p=0.000). Post-hoc analysis using Mann-Whitney test showed significant difference in all years except year 1 and 4 (p=0.388). Of the 9 lecturers interviewed during the second interviews, 7 endorsed the importance of CI, while 2 doubted its applicability.

**Conclusions:**

Due to the unique characteristics of each block, our system had not been able to conduct a balanced CI evaluation, as compared to the original research. We concluded that the implementation of CI would be highly dependent on the faculty members, with their commitment as the main pre-requisite. We hope to involve academic advisors as CI evaluators and improve our student-centered learning for future assessments. Further study is needed to investigate the longitudinal implementation of CI.

## Introduction

Assessing medical students’ professionalism often raised both global and national discussions. The National Practitioner Data Bank (NPDB), Medical Malpractice Payment Reports (MMPR), and Adverse Action Reports (AAR) reported 599,945 of total malpractice cases in the U.S. on all practitioners for the years 2004–2014.^1^ In Indonesia, since 2006 to 2012 there were at least 182 documented cases of malpractice in the medical field.^2^ There are evidence that negative student behaviors during undergraduate programs are related to the likelihood of the similar behaviors in later careers.^3^^-^^4^ This emphasized the need to develop an effective measurement of such behaviors, particularly one that could be implemented early in pre-clinical years. Academic medicine has described professionalism mostly in more of value-based manners,^5^ which encompass attributes beyond the application of knowledge and skills: to embrace humanism, integrity, accountability, altruism, ability to work in a team, the pursuit for excellence, and many others. Difficulties in assessing professionalism may be attributed to the complex qualitative nature of professionalism. Thus, a powerful measurement for professionalism remains indeterminate.^6^^-^^9^

The student evaluation process should reflect the importance of demonstrating professional behaviors. Many current methods of assessing professionalism rely on multiple assessments of an individual’s professional behaviors taken over a period of time.^9^ McLachlan, et al. developed a quantitative measurement of professionalism, called Conscientiousness Index (CI), which records spontaneous behaviors. CI is a valid, reliable, and effective measurement using simple award or deduction of specified CI points for each student.^3^ Such measurement is expected to help identify individuals that will be more likely to display unprofessional behaviors in their practice later, and therefore, its use in pre-clinical years will permit possible interventions to support such individuals.^6^

The purpose of the study was to design a pilot study to apply CI at the School of Medicine, Atma Jaya Catholic University of Indonesia (SMAJCUI). We collected the data from our school and year 1 – 4 pre-clinical medical students who joined voluntarily in this research. We would like to gain faculty members’ perspectives about suitable CI components and implementations during pre-clinical years at the School of Medicine, AJCUI. In addition, we analyzed the difference of CI scores in year 1 – 4 students.

## Methods

### Study design

**Figure 1:**
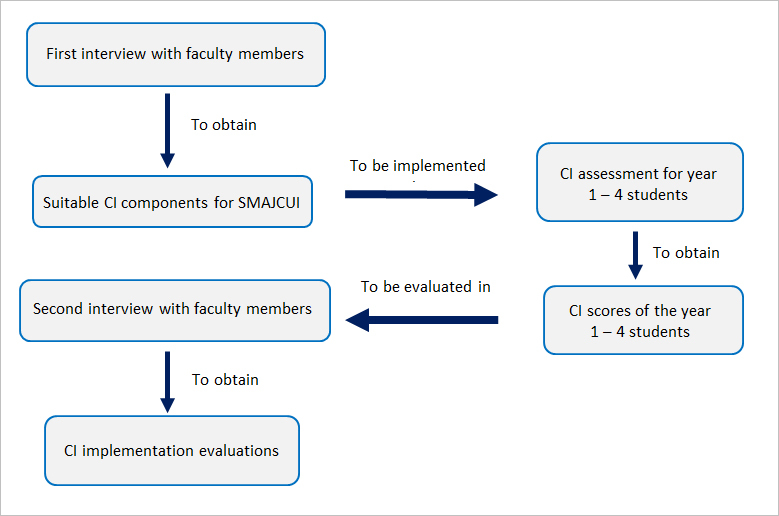
Study design

This research combined both qualitative and quantitative studies. The qualitative study consisted of two series of interviews. The first interviews were conducted to acquire the faculty members’ perspectives about the suitable CI components for SMAJCUI. The second interviews were conducted to acquire the faculty members’ perspectives about CI implementation at SMAJCUI.  The quantitative study consisted of the CI assessment. We held our study during the first semester of the academic year 2014–2015. We explained about the purposes and process of the study to our students verbally in a class session at the beginning of the semester, and distributed informed consent forms in problem-based learning (PBL) sessions. Our student participants were purposively selected from the PBL groups that had submitted their informed consents. Ethical approval was obtained from the Ethical Committee of School of Medicine, Atma Jaya Catholic University of Indonesia (on 23th July 2014). Our study design was briefly described in Figure 1. Faculty members from certain blocks were asked about the data they recorded which led to the award and deduction of CI points. Students received 50 points as starting score. The conscientiousness points are summarized below.

#### Attendance

We used the records from the compulsory attendance of Problem-Based Learning (PBL) and Clinical Skills (CS) sessions. The attendance records were collected from tutors and administrators. Students will receive deduction by 1 point if they were absent without permission or late for more than 10 minutes.

#### Adherence to rules

Adherence component was divided into adherence in didactic, CS, and laboratory classes. Students received 1 point deduction for each violation of specific rules in these classes (e.g. students were ought to use appropriate clothing).

#### Evaluative feedback submissions

We recorded evaluative feedbacks from PBL, CS, Peer-Assisted Learning (PAL), lecturers, and the Dundee Ready Educational Environment Measure (DREEM) evaluations. Students were awarded 1 point for each evaluation submitted. We collected the data from faculty members, school administrators, and DREEM researcher. Each block had a different number of evaluative feedbacks.

#### Performance in assignments and clinical skills

Students received 1 point deduction if their assignment or CS examination was deemed unsatisfactory to faculty members’ criteria. We collected the data from faculty members; including block directors and CS tutors. The number of assignments and clinical skills varied between blocks.

#### Assignment submissions

Students received 1 point for each assignment submitted on time. Students who failed to submit on time or did not submit received no points. All assignments contributed to the decision.

#### Volunteerism

Students who participated in voluntary activities received additional 1 point for each participation.

#### Accomplishments

Students received additional 1 point for each excellent accomplishment or award (e.g. winning competition or becoming organization committee).

#### General misconducts

Students received deduction of points for each misconduct in our school environment. These unacceptable behaviors include smoking, scuffle, plagiarism, and drug misuse in campus area.

#### Study participants, sample size, and sampling methods

We conducted a series of first interviews with faculty members (N=12) from May to June 2014, using a purposive sampling method. The twelve participants were nine medical doctors and four non-doctors. We selected these faculty members using inclusion criteria: (1) Experienced in teaching medical students, (2) Had the capability as a mentor from past experiences and recommendations, (3) Had close–repeated contacts with students in teaching or other academic-/campus-related activities through a period of time, and (4) Had participated in the blocks where CI evaluation was conducted.

The first to fourth year pre-clinical medical students (N=144) participated as our student participants; ranged from 31–38 students for each year. We collected our data from one block for each year; the first year from Biomedical Science I block, the second year from Concepts of Pathology I block, the third year from Neuromusculoskeletal block, and the fourth year from Addiction Medicine elective. We purposively selected these blocks due to our collaborations with the faculty members contributing in the interviews.

We held the series of second interviews with the chosen faculty members (n=9). Three faculty members (one from Department of Pharmacology, one from Department of Biomedicine, and one from Department of Neurology) were not included, because of their insignificant contribution to the blocks where the CI evaluation was conducted.

### Data analysis methods

We used SPSS version 20.0 to analyze the CI scores. Kolmogorov-Smirnov test was conducted, followed by Kruskal-Wallis test and post-hoc analysis using Mann-Whitney test. We conducted content analysis using Weft-QDA to manage the verbatim transcripts for the qualitative results. Our research team carried out five inter-rater meetings to obtain inter-rater reliability for data dependability and qualitative analysis.

## Results

### Quantitative results

CI scores ranged from 61 to 64 in year 1 students batch, 53 to 61 in year 2 students batch, 55 to 69 in year 3 students batch, and 58 to 61 in year 4 students batch. We converted these scores into percentages of the maximum possible scores in each batch. Kolmogorov-Smirnov test showed an abnormal distribution across the CI scores from year 1 – 4 pre-clinical medical students (p=0.005). Kruskal-Wallis test showed a significant difference in CI scores between each year (p=0.000). We then carried out the post-hoc analysis using Mann-Whitney test, which showed significant differences between all year-to-year comparisons (p=0.000), except the comparison of year 1 and 4 CI scores (p=0.388).

### Qualitative results

We used the probing technique for the interviews. We created a modified list from McLachlan’s CI components as a trigger for the faculty members to revise, add or delete some of the components if necessary. Faculty members were also asked to describe medical professionalism and its assessment.

Questions asked at the second interviews were listed below:

1.        What is your opinion about CI scores’ evaluation?

2.        Do you think that the CI can be applied at SMAJCUI?

3.        What are the CI components that can be used to assess professionalism at SMAJCUI? Why?

4.        What are your suggestions regarding the development of CI instrument in the future?

Fourteen themes were identified based from the first and second series of interviews. The following description of the themes was the summary of what the faculty members expressed, illustrated by selected quotations and translated as closely as possible from Bahasa Indonesia to English.

#### Medical professionalism

Faculty members described medical professionalism as the way doctors carried their profession with good competence, especially in terms of their clinical service. Some faculty members emphasized medical professionalism on communication skills.

“Professionalism, in general, is described as a good competence demanded by society based on the education of that person’s profession.” (Faculty member 4, first interviews)

“… in these components, we are not only emphasizing on knowledge and cognitive skills of the students’, but also their communication skills; with other colleagues, teams, and patients.” (Faculty member 11, first interviews)

One faculty member described that one of the distinct aspect in medical professionalism was that doctors ought to put the patients’ interest over theirs.

“… what distinguishes doctors from other professions, is that we must put our patients’ interest above ours.” (Faculty member 5, first interviews)

### Assessment of medical students’ professionalism

During the first interviews, we found that five faculty members were unable to name any professionalism assessment tools. Seven others described: legal standards, behaviors assessments, Objective Structured Clinical Examination (OSCE), Clinical Skills (CS), and a 360-degree evaluation.

Some faculty members described professionalism as a wide spectrum of behaviors, as presented in CI. In the first interviews, two faculty members even planned to track professional behaviors of their students (e.g. from the students’ attendance and assignments).

“… we planned to make a learning contract; in which we are going to include students’ attendance in 5% of their total block score … We will require students to be present on time and complete the given tasks.” (Faculty member 6, first interviews)

“… This time, I am planning to make an evaluation in my block. Daily evaluations … for instance, whether they have submitted their assignments on time … whether they have come on time …” (Faculty member 11, first interviews)

#### Conscientiousness Index

Three out of twelve faculty members questioned the concept of CI and whether CI was a standardized evidence-based assessment. One faculty member recognized that not all CI sub-components could be assessed in his/her block, including PAL, personal assignments, and laboratory studies.

#### Attendance

During the first interviews, a faculty member questioned the PBL and CS tutors’ punctuality. Our collaboration with PBL and CS administrators was important to achieve appropriate data collection. Faculty members suggested that PBL and CS attendance records would be more easily obtained than class attendance records due to the school’s regulation.

“We can use the attendance to class, PBL, and CS sessions as data, these are available in each block. But there are no regulations that require students to attend a specific number of class session to pass the block, so the only viable options are PBL and CS attendance.” (Faculty member 1, second interviews)

#### Adherence to rules

Our faculty members asserted that the adherence to rules could be used as a CI component. We modified the adherence component into three major sub-components; adherence to didactic teaching (e.g. to use appropriate clothing and to not eat during classes), CS (e.g. to use appropriate clothing and keeping nails clean), and laboratory rules.

#### Evaluative feedback submissions

The evaluative feedbacks in our school varied between blocks. Most of the evaluations were written on papers, except for lecturer and peer tutor evaluations. Faculty members were concerned about the anonymity of the feedbacks that would result in difficulties tracking CI data records. Moreover, our faculty members were concerned that some of our students might have completed the evaluations without putting adequate thoughts into them.

“… it should be noted that some students will fill in those feedback forms carelessly. Is there any standard for this? …” (Faculty member 3, first interviews)

From the results, we acknowledged that evaluative feedbacks from PBL and CS could not be obtained, due to the anonymity of the written evaluative feedbacks. Meanwhile, PAL evaluations were done only during the first year. A faculty member described the importance of collaborating with other facilitators, so that we would receive the full data of evaluative feedbacks for further studies.

### Performance in assignments and clinical skills

Four faculty members argued that it would be hard to assess the professionalism of individual students, when most of the academic assignments were given as group projects. During the second interviews, a faculty member suggested the use of more individual-based tasks to help assess the personal aspect of professionalism.

“… There should be more individual projects, not only group projects. It needs to be more specific …” (Faculty member 7, second interviews)

#### Assignment submissions

We had planned to measure the submission of individual assignments. However, tasks and projects were more frequently assigned to groups rather than individuals. Thus, we decided to include group assignments as well, considering that individual responsibility should also be involved in such assignments. A faculty member even described that it was hard to ask for each block to give individual projects.

“… I think it would be rather difficult to assign individual projects in every single block. Perhaps we should consider other options …” (Faculty member 12, first interviews)

A faculty member suggested the submissions through Moodle™ to facilitate data collection.

“We could use Moodle™ … we could give information about assignments, deadlines, and make the data collection process much easier …” (Faculty member 11, first interviews)

A faculty member advised to compare the amount of tasks given in different years.

“… Perhaps [CI score] can be measured through a range of scores, in percentages. Since we don’t have the same number of assignments in each block … For example, from 10 assignments, he did 7 assignments, so he got 70% of the score … In the other block, there were only 5 assignments, he did 3, so it’s basically a same score, right?” (Faculty member 9, second interviews)

#### Volunteerism

Some faculty members described voluntary activities as those associated with medical professions, such as community health services. Others explained that voluntary activities might also include: becoming a research assistant or a peer tutor in PAL.  A faculty member admitted that it would be hard to observe students’ activities outside our school, if we used students’ senate as the information source. Therefore, most of the personal information for voluntary activities and achievements were enquired from our students through thought exchanges that occurred during periodic meetings between the faculty member and the students.

#### Accomplishments

Our faculty members suggested direct contact with the medical students as a way to record student accomplishments.

“… You can use a personal approach to record the data from our students. You can talk to them …” (Faculty member 7, first interviews)

One of our faculty members asserted the need to ensure every student to excel.

“… If we can ensure the excellence of our students, in the future we will end up with doctors who not only excel in clinical aspects but also respect the adherence to rules and have the capability to think outside the box, with creativity and initiative …” (Faculty member 8, second interviews)

#### General misconducts

No faculty members reported any violation to rules. During the first interviews, a faculty member predicted that it would be hard to record any misconducts. Despite the obstacles, some faculty members claimed that they tried to assess general misconducts in their blocks.

“We could make a personal assignment for each student, and then we compare them. We would know if someone just ‘copy-pasted’ their assignments… Then, we’ll evaluate it.” (Faculty member 11, first interviews)

A faculty member argued that it was hard to judge plagiarism as a misconduct.

“… There were a lot of people who do not know about plagiarism … There should be a common understanding explained through seminars … if we have not educated people about plagiarism, accusing students of doing so might be unfair to them … Then we can ask them to make an anti-plagiarism contract …” (Faculty member 3, first interviews)

#### Suggestions

Respect for colleagues was one of the suggested components that included identifying bullying behaviors towards other students. Most faculty members recognized the importance of collaborations between all faculty members to integrate CI at the SMAJCUI (e.g. dean, block leaders, and medical education unit).

“… We need collaboration with block leaders, 1st Vice Dean and medical education unit … we will come up with a standard …” (Faculty member 8, second interviews)

Faculty members also suggested the importance of training and socialization of CI.

“… faculty members should be trained to perform professionalism assessment … This assessment should be integrated in each block …” (Faculty member 11, second interviews)

A faculty member suggested the use of other professionalism assessments in pre-clinical and clinical settings. This described the importance of professionalism assessment to ensure the growth of medical students.

“We haven’t been able to design an assessment that could monitor the growth of every student … if we had a small group of students, it would be much easier to assess and interact with them … in pre-clinical settings, especially in big classes, it’ll be harder … In clinical settings, we will see more of these processes … for further studies, perhaps we could try to apply this scale in clinical settings.” (Faculty 11, second interviews)

#### Evaluations

Reactions from our faculty members varied. Some might deem CI as an unsuitable scale for SMAJCUI, but other faculty members agreed that the results portrayed professional behaviors of medical students. CI could be used as an input to our education system.

“… from my perspective, CI is not really specific or accurate … if we compare CI from all batches; they had different lectures, different blocks. There would be a lot of differences …” (Faculty member 7, second interviews)

“… At first, I was shocked by the results … As a lecturer, I should be asking, why? … this could be an input to the faculty members and our institution …” (Faculty member 8, second interviews)

“… Before the assessment started, I already predicted that we would be lacking in the similarity between blocks (e.g. the feedback components, etc.). I think that these results were reasonable …” (Faculty member 3, second interviews)

#### Applicability

Seven out of nine faculty members agreed that CI assessment could be applied at our school. Most faculty members considered the importance of school policies to support CI implementation (e.g. socialization of CI assessment and a more complete data records).

“… It is important for an educational institution to help students to become professional individuals. It’s our [faculty members] duty to think about ways to implement CI … and if you ask me, whether or not CI is applicable: Yes, it is! … But perhaps we need further discussions and evaluations from each block …” (Faculty member 8, second interviews)

A faculty member argued that the shortage of faculty members at SMAJCUI could be one of the obstacles in future CI implementation.

“Yes, it would be hard to remodel the current system, but it’s possible. From my perspective, the obstacle would be the number of students compared to faculty members …” (Faculty member 11, second interviews)

To ensure the dependability and transferability of our qualitative data, we performed inter-rater meetings and an audit trail. The inter-rater agreement for the first interviews shifted from 21% to 93.6%. For the second interviews, the inter-rater agreement shifted from 20.9% to 95.8%. We continued with an audit trail with the help of an external auditor. The auditor agreed that our data was valuable and significant to conclude the study.

## Discussion

This study employed both qualitative and quantitative approaches to explore the possibilities of CI implementation. Unlike previous researches3, this study showed that there was a significant difference between CI scores in almost all years, except year 1 and 4. Our discussion will mostly emphasize on the differences of students’ scores, faculty members’ perspective of CI, and study limitations.

### Differences of students’ scores

The post-hoc analysis showed significant differences almost in all years, except for year 1 and 4. We found higher median of CI scores in year 1 and 4 (97% and 95%), compared to year 2 and 3 median scores (83% and 92%).

We predicted the number of assignments from each block as a major contributing factor in the significant difference between the CI scores; year 1–4 medical students received 9, 2, 4 and 6 assignments, respectively. All of our students collected their assignments. Assignment completion could be one of the main strength in assessing professionalism.^10^ Considering this fact, a more reliable assessment such as individual assignments should be assigned to allow possibilities of a better individualized assessment.

Faculty members from Biomedical Science I (year 1) and Addiction Medicine (year 4) managed their block creatively through student-centered learning (e.g. peer-assisted learning and field studies). Assignments listed in Biomedical Science I block were: a concept map project (individual task), a creative assignment (group task), and 7 quizzes (individual task). Assignments listed in Addiction Medicine block (year 4) were: field studies to the National Narcotics Enforcement Agency and a substance abuse treatment center (group task), jigsaw assignments (group task), cased-based learnings (group task), focus groupdiscussions (group task), a creative health promotion (group task), and a creative assignment (group task). Assignments given in Pathology Concept I block (year 2) were: projects on HIV/AIDS topic (group task) and the effects of aging in immune system (group task). Assignments given in Neuromusculoskeletal block (year 3) were: case-based discussions (group task), project on pain managements (group task), and two quizzes (individual task).

The highest raw score of year 3 students was 69 and the median score was 57. Volunteerism and accomplishments were the major contributing components in the large gap between these scores. Students who volunteered in social activities, joined organizations and event committees, or participated in medical competitions received higher CI scores compared to their colleagues who did not. We believe that these components serve as an important factor in comparing the professional attributes between individuals.^7^^,^^8^^,^^11^

### Faculty members’ perspective of CI

Some faculty members changed their opinions regarding the implementation of CI in the medical school education system. During the first interviews, all faculty members agreed on the importance of CI as a professionalism assessment tool. After the second interviews, only 7 out of 9 faculty members were able to suggest the benefits of using CI.

Some claimed that the concepts of plagiarism were not familiar to the students. In truth, plagiarism, that has been correlated with unprofessional traits and failures in personal integrity,^12^is one of the main academic violations in SMAJCUI. Our study emphasized the need for faculty members’ commitment and understanding about professionalism.

The interviews revealed lack of understanding from some faculty members regarding the concept of professionalism; including the shift of professionalism towards values, attitudes, and especially behaviors.^8^^,^^11^^,^^13^ Moreover, during the first interview, some faculty members admitted the lack of understanding regarding professionalism assessment. One faculty member even misunderstood CI as a questionnaire. With these notions, we would like to advocate the importance of commitment from faculty members themselves to assess medical students’ professionalism, as doctors who possess an inseparable responsibility as teachers.

Some faculty members recognized that CI should not be compared between different years due to the varied and diverse programs in each block at SMAJCUI. The primary challenges for CI would be for faculty members to socialize and implement CI in their blocks. Thus, a supportive environment, such as role models, will be important to help students grow into their professional self.

Implementation of CI requires a longitudinal assessment.^3^^,^^6^^,^^9^^,^^10^^,^^14^ One faculty member advised the importance of a longitudinal assessment to improve professional attributes of future doctors. We believe that academic advisors’ roles will benefit the implementation of CI at SMAJCUI. An academic advisor is a faculty member assigned to guide students from the beginning of their admission to their graduation from medical school. Academic advisors will be able to provide supervision for students’ professional behaviors, especially in the three major components: volunteerism, accomplishments, and general misconducts. The other five components can be assessed by block teams. We also considered the importance of rewards and incentives for academic counselors and faculty members to compensate for their increased workload by their participation in CI assessment and evaluation.^15^

### Study limitations

Our study had a number of limitations. The CI scores collected were unbalanced between each year due to unique characteristics of each block. Most of the assignments were given in groups with only a few personal assignments from Biomedical Science I and Neuromusculoskeletal block. Professional behaviors of each individual might be reflected less accurately by group assignments. For further studies, we advised faculty members to give at least one personal assignment during each block for the formative assessment and component scoring.

Our study had 31 – 38 student participants per year and only one block was selected for each year. For future studies, we suggest a comprehensive socialization and participation of all faculty members, block contributors, and medical students.

There were difficulties in obtaining a complete record on teaching and learning components in our school. There might be recall biases regarding our data records, because some of our data were taken by enquiring students and faculty members. Thus, we valued the importance of managing student-centered learning (SCL) in which students may have direct feedback from their tutors (faculty members). Our school have applied PBL, CS, CBL, PAL, and e-learning, but only PBL and CS were fully applied in all blocks. Records on assignments and feedback through the SCL need to be managed in the way students contribute to their own records (peer-assessment) and tutors responsible to report the records (tutor assessment) to the managing director.

E-learning could be used as a media to facilitate learning between students and faculties.^16^ McLachlan, et al. used e-learning in the form of Virtual Learning Environment.^3^^,^^6^^,^^9^ Full implementation of e-learning would benefit the system as well as CI data recording (evaluative feedback submissions, volunteerism, accomplishments, and general misconducts). This was possible, due to the fact that our school had already collected faculty members’ evaluative feedbacks using e-learning. Several studies had concluded that some students preferred online evaluations rather than written ones.^15^ The Biomedical Science I block also used e-learning to collect PAL evaluative feedbacks and assignments. E-learning would clearly be beneficial to our data records. For future studies, we recommended the use of e-learning.

One faculty member claimed that one of the obstacles in applying CI was the limited number of faculty members. From our data, SMAJCUI had the ideal ratio between faculty members and students (1:8). If that is not enough, we predicted that integrating peer-assisted learning (PAL) would be beneficial as a short-term solution. PAL was one of the way for students (tutors and tutee) to learn and practice their communication skills.^17^^,^^18^ PAL program integration might add more workload to the faculty members (e.g. recruiting, training, and monitoring peer tutors); however, as shown in the Biomedical Science I block, the method was proven to be successful. Routine evaluations, assignments, and quizzes could also be used to help monitor and evaluate the implementation of future PAL program.^19^

## Conclusions

Medical institutions and faculty members need to explore professionalism of their medical students as early as in pre-clinical years.^3^^,^^8^^,^^13^^,^^20^ There was no exact method or theory that could be used as a way to assess professionalism in the curriculum. The only exact understanding is that every academic institution should ensure their graduates’ professional qualities.^21^

Although 7 out of 9 faculty members supported the use of CI, its implementation would certainly need faculty members’ collaborations, as well as their support and commitment to assess their students’ professionalism. From our research, we concluded that CI is highly dependent on the faculties. Due to the unique characteristics of each block, our system had not been able to conduct a balanced CI evaluation, as compared to the original research.3 We concluded that longitudinal, time-to-time evaluations are essential, using various approaches to the study of professionalism, including CI.^8^^,^^11^^,^^22^

### Acknowledgements

The source of financial grants for this research came from the School of Medicine, Atma Jaya Catholic University of Indonesia. We would like to thank Ratnawati Linarto and Janet Apriyani for their help as the inter-raters of this research, Nurul Itqiyah Hariadi and Christopher The as the editors of this article, and Dhevy Setya Wibawa as the external auditor, as well as the faculty members, administrators, and medical students who helped this research become possible.

### Conflict of Interest

The authors declare that they have no conflict of interest.
